# Is Piezosurgery Associated with Improved Patient Outcomes Compared to Conventional Osteotomy in Rhinoplasty? A Systematic Review and Meta-Analysis of RCTs

**DOI:** 10.3390/jcm13133635

**Published:** 2024-06-21

**Authors:** Alaa Safia, Uday Abd Elhadi, Raed Farhat, Salman Elgrinawi, Jawad Safieh, Rawnk Bader, Ashraf Khater, Shlomo Merchavy, Saqr Massoud

**Affiliations:** Head & Neck Surgery Unit, Department of Otolaryngology, Rebecca Ziv Medical Center, Safed 1311001, Israel; udayabdi510@gmail.com (U.A.E.); raed.frhat88@gmail.com (R.F.); salmanalkro999@gmail.com (S.E.); safeyejawad@gmail.com (J.S.); rawnaq1605@gmail.com (R.B.); dr.akhater@gmail.com (A.K.); shlomo.m@ziv.health.gov.il (S.M.); saqr.m@ziv.health.gov.il (S.M.)

**Keywords:** piezosurgery, conventional osteotomy, rhinoplasty, edema, ecchymosis

## Abstract

**Background:** Rhinoplasty is a common plastic surgery procedure with evolving surgical techniques. This systematic review and meta-analysis compares the outcomes of piezosurgery versus conventional osteotomy in rhinoplasty. **Methods:** A comprehensive search of six databases yielded 12 randomized controlled trials (RCTs) comparing piezosurgery (292 cases) to conventional osteotomy (338 cases) in rhinoplasty patients. The examined outcomes included postoperative edema, ecchymosis, complications, pain (using the Visual Analogue Scale—VAS), and operative time. Subgroup analyses were conducted based on the assessment timepoint, surgical approach, and outcome grade. The risk of bias was evaluated using the revised Cochrane tool. **Results:** Piezosurgery showed a significant reduction in the degree of postoperative edema (second and seventh postoperative days) and ecchymosis (second, fourth, and seventh postoperative days). The external approach in piezosurgery demonstrated greater benefits for both outcomes. Piezosurgery was associated with a significant reduction in overall complications, especially mucosal injuries, compared to conventional osteotomy, with no significant difference regarding postoperative hemorrhage. A significant reduction in pain scores and the need for analgesia was observed with piezosurgery. No significant difference was found in operative time. **Conclusions:** Piezosurgery offers significant benefits in patient outcomes, with similar operative time between both techniques. However, long-term investigations are still needed.

## 1. Introduction

Rhinoplasty, one of the most frequently performed plastic surgeries worldwide, aims to alter the functional and aesthetic aspects of the nasal structure [1]. While traditional methods have proven effective, the advent of piezosurgery has introduced a new dimension to this field [2]. Piezosurgery, a technique utilizing ultrasonic vibrations for bone cutting, promises precision and minimal invasiveness, potentially revolutionizing rhinoplasty outcomes [3].

Traditional rhinoplasty techniques, primarily based on conventional osteotomy, have been the cornerstone in nasal surgeries [4]. These techniques, while effective, are often associated with significant postoperative complications such as edema, ecchymosis, and prolonged pain [5]. With the increasing demand for less invasive and more precise surgical interventions, piezosurgery has emerged as a promising alternative. Its application in rhinoplasty is a reflection of ongoing efforts to refine surgical techniques to improve patient outcomes and satisfaction.

Piezosurgery, utilizing high-frequency ultrasonic oscillations, allows surgeons to cut bone with remarkable precision while sparing soft tissue [6]. This technology is theorized to reduce the trauma associated with bone manipulation, potentially minimizing postoperative complications such as edema and ecchymosis and reducing pain. However, despite its theoretical advantages, the clinical superiority of piezosurgery over conventional osteotomy in rhinoplasty remains a subject of debate [6].

Given the conflicting reports and varied clinical practices [5,7,8], a systematic comparison of piezosurgery and conventional osteotomy is essential. By synthesizing data from randomized controlled trials (RCTs), a clearer understanding of the comparative effectiveness and safety of these techniques can be established. This is vital for guiding clinical practice and informing patient choices. Various systematic reviews have been conducted in this regard; however, their questionable methodology and small sample size limited the interpretation of the provided evidence (Table 1) [9,10].

This systematic review and meta-analysis aimed to compare piezosurgery with conventional osteotomy in patients undergoing rhinoplasty. Specifically, it focuses on evaluating postoperative outcomes such as edema, ecchymosis, complications, pain, and operative time. By providing a comprehensive analysis of these outcomes, this study seeks to contribute valuable insights into the optimization of surgical techniques in rhinoplasty.

## 2. Materials and Methods

### 2.1. Design and Population

This research was conducted in line with the guidelines provided for systematic reviews and meta-analyses in the Preferred Reporting Items for Systematic Reviews and Meta-Analyses (PRISMA) checklist. The study protocol was registered on PROSPERO (CRD42023490526). The design of this review followed the PICOS (Population, Intervention, Comparison, Outcome, and Study) framework [12]. We included patients undergoing rhinoplasty. The intervention group included rhinoplasty using piezosurgery, while the comparison (control) group included rhinoplasty through conventional osteotomy. The main outcomes included postoperative edema, ecchymosis, pain, complications, mucosal injury, and hemorrhage as well as operative time. Only randomized controlled trials (RCTs) were considered.

### 2.2. Literature Search

On 1 November 2023, a literature search was performed across four databases (PubMed, Scopus, Web of Science, and Cochrane Registry of Randomized Controlled Trials) and two registries (clinicaltrials.gov and Google Scholar). In the latter, as per recent guidelines, only the first 200 records were selected and screened. A list of relevant keywords and medical terms was used, which were adjusted accordingly as per the searched database. These terms included “rhinoplasty”, “osteotomy”, “piezosurgery”, and “random.” The full search query, adjusted per the searched databases, is provided in Table S1. Additionally, a manual search was conducted to ensure the quality of the performed database search/screening as well as to identify any other relevant, potentially missing studies. This step was conducted by reviewing the relevant review articles on this topic, screening the reference list of included studies in our review, and searching for relevant articles to finally select ones through the “similar articles” function on PubMed [13].

### 2.3. Selection Criteria

Studies meeting the following criteria were included: (a) randomized controlled trial; (b) investigated rhinoplasty; (c) patients were allocated to piezosurgery (intervention) and conventional osteotomy (control); and (d) reported one of our main outcomes. We did not put a limitation on the language of original publication nor the date of publication.

The exclusion criteria included the following: (a) single-armed studies “lack of a comparison group”, (b) non-randomized studies of intervention, (c) secondary research papers (i.e., reviews, opinions, commentaries, etc.), (d) protocols, (e) irrelevant outcomes, and (f) duplicated papers.

### 2.4. Outcomes Measures

The primary outcomes of interest included postoperative edema and ecchymosis. The secondary outcomes of interest included postoperative pain, pain requiring analgesia, complications, operative time (hour), nasal airflow, and odor function. The postoperative pain level was measured using the Visual Analogue Scale (VAS); subjective pain was not considered for valid assessment. Pain requiring analgesia was defined as a VAS score >3 [14]. Complications were divided into overall complications, mucosal injury, or hemorrhage or epistaxis. Effect modification based on the surgical approach (external vs. internal osteotomy), outcome grade (for edema and ecchymosis), and assessment timepoint was investigated.

### 2.5. Study Selection

Studies identified from the literature search were imported into EndNote software (Version 8), where duplicates were identified and excluded. After that, the remaining studies were exported into an Excel sheet for screening. The screening process was performed by two investigators simultaneously over three different phases: title, abstract, and full-text selection. Any differences between them were resolved by consulting the senior author.

### 2.6. Data Curation

The senior author used Microsoft Excel to design the data extraction sheet, which consisted of three worksheet tabs. The first part covered studies’/patients’ characteristics, including studies identification number, authors names, year of publication, country of investigation, study design, sample size, age, gender, surgical approach, osteotomy type, and the type of instrument used. The second part of the sheet covered our outcomes of interest (edema, ecchymosis, pain, complications, operative time, nasal airflow, and odor function). The third part covered the methodological quality (risk of bias—ROB) of the included studies.

### 2.7. Methodological Quality Assessment

The revised Cochrane RoB-II tool (revised in 2019) was used to assess the methodological quality of the included trials [15]. Each RCT will be assessed on the level of five domains: randomization, deviation from intended interventions, missing outcome data, outcome measurement bias, and selective reporting. Each RCT will be given a quality label of a low risk of bias, some concerns, or high risk of bias.

### 2.8. Data Synthesis and Analysis

The data analysis was performed using STATA Software (Version 18) according to the analysis plan a priori. Outcomes (nasal airflow and odor function) were not quantitatively analyzed because they were only reported by one trial [16]. A pooled meta-analysis was performed by pooling the log odds ratio for binary outcomes and the mean difference for continuous outcomes. The random-effects model was used when a significant level of statistical heterogeneity was observed, defined as I^2^ > 50% with a *p*-value < 0.05. Statistical heterogeneity was categorized as follows: non-important (0–40%), moderate (30–60%), substantial (50–90%), and considerable (75–100%). Notably, a pooled meta-analysis of postoperative edema, ecchymosis, and pain was not feasible as the degree of these outcomes differed by time; thus, the main analysis was a subgroup analysis based on the assessment timepoint. When significant heterogeneity was identified, a combined approach was attempted using leave-one-out sensitivity analysis performed to determine if the reported effect estimate was driven by a particular study. Subgroup analyses were performed based on time, surgical approach, and edema/ecchymosis grade. The assessment of publication bias was not feasible because all analyses included an insufficient number of trials (<10 RCTs).

## 3. Results

### 3.1. Literature Search and Screening Results

**Figure 1:**
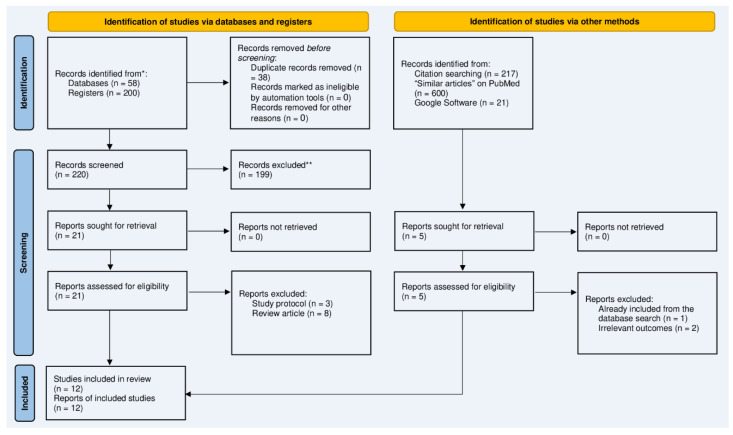
PRISMA diagram showing the results of the literature search and screening steps. * The following databases were searched: PubMed, Scopus, Web of Science, and Cochrane Registry of Randomized Controlled Trials; ** indicates the results of the title and abstract screening phases.

The results of the database search and screening processes are illustrated in Figure 1. In summary, the database search yielded 58 articles, while 200 records were retrieved from Google Scholar. Of 258 retrieved citations, 38 duplicates were identified and removed with EndNote software (Version 8). The title/abstract screening of 220 resulted in 21 articles eligible for full-text screening. All full texts were accessed, of which 11 were excluded: 3 were study protocols and 8 were review articles. The manual search resulted in five articles, of which two were highlighted as additional, relevant articles [14,17]. Eventually, 12 RCTs were quantitatively synthesized [5,7,8,14,16,17,18,19,20,21,22,23].

### 3.2. Baseline Characteristics of Included Studies

Most evidence was provided from Turkey (five RCTs) followed by Iran (three RCTs), Egypt (one RCT), Austria (one RCT), Italy (one RCT), and the United States (one RCT). Twelve RCTs allocated patients to either piezosurgery (292 patients) or conventional osteotomy (338 patients). Of note, in two RCTs, the same patients accounted for both the intervention and control based on the operative side [17,20]; and thus, analyzed data were based on the operated sides not the number of cases. The male-to-female ratio was 1: 2.05. Most patients were young adults. A detailed description of the operative approach (internal or external osteotomy), the type of osteotomy, and the instruments used in each group is provided in Table 2.

### 3.3. Methodological Quality

**Figure 2:**
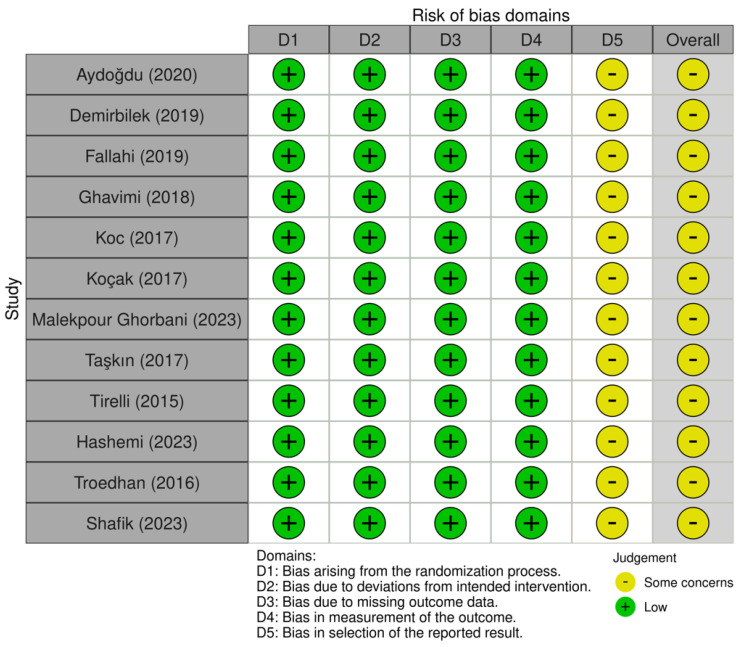
A diagram showing the risk of bias of included randomized controlled trials through the revised Cochrane tool [5,7,8,14,16,17,18,19,20,21,22,23].

The detailed assessment of the risk of bias on included RCTs is illustrated in Figure 2. In summary, all of the included RCTs had some concerns, seldomly related to the “selection of reported results” domain. This is because none of included RCTs had publicly available protocols that could be accessed to determine whether or not the authors adhered to the analysis plan a priori.

### 3.4. Postoperative Edema

#### 3.4.1. Degree of Edema

**Figure 3:**
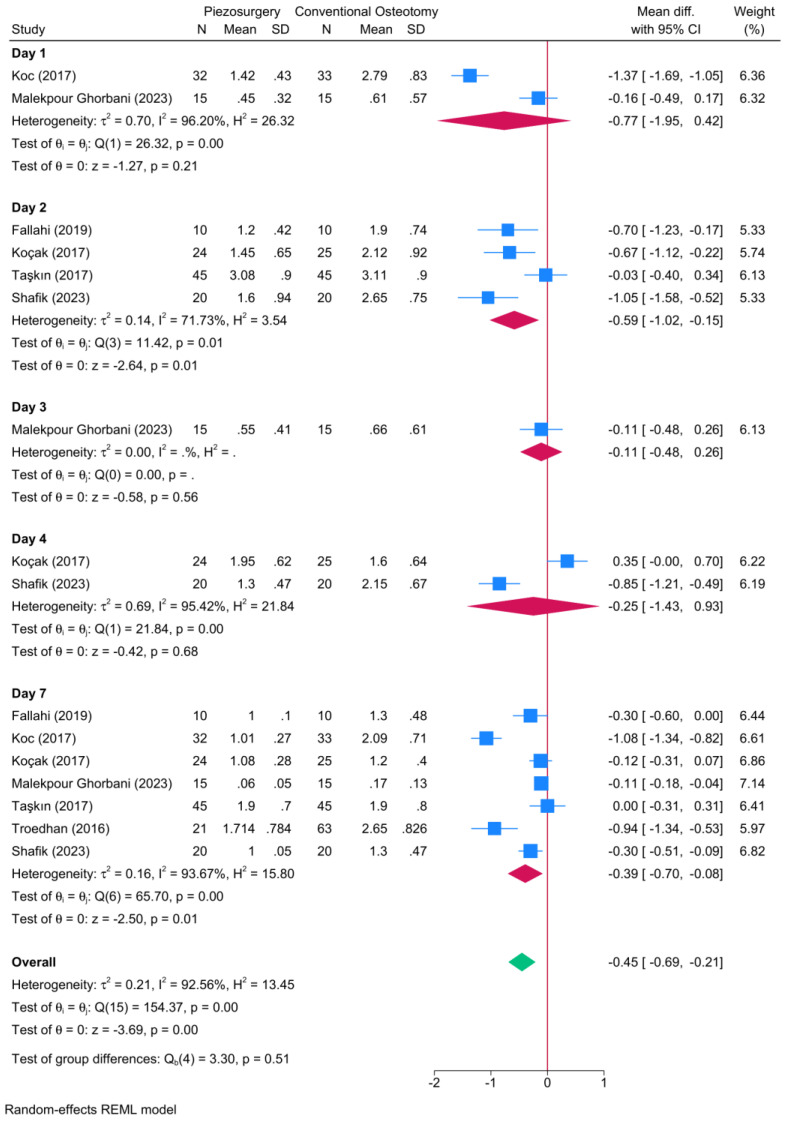
Forest plot showing the difference in the degree of postoperative edema between piezosurgery and conventional osteotomy in rhinoplasty patients [5,7,8,14,20,21,23]. Blue squares reflect the effect size of individual studies, while the red rhombi indicate the pooled effect size per each subgroup. The green rhombus highlights the overall pooled effect estimate across all subgroups.

Seven RCTs reported data regarding postoperative edema (Figure 3). A significant reduction in the degree of postoperative edema was observed in favor of piezosurgery on the second [four RCTs, MD = −0.59; 95% CI: −1.02: −0.15] and seventh postoperative days [seven RCTs, MD = −0.39; 95% CI: −0.70: −0.08]. No significant different was noted between piezosurgery and conventional osteotomy during the first, third, and fourth postoperative days. 

#### 3.4.2. Risk of Edema

The risk of postoperative edema was reported by four RCTs (Figure S1). The pooled meta-analysis showed no significant difference in the risk of edema between both surgeries [logOR = −1.19; 95% CI: −2.46: 0.08]. However, a moderate level of heterogeneity was evident [I^2^ = 70.94%, *p* = 0.01]. The subgroup analysis based on the assessment timepoint showed no difference except for Day 3 [logOR = −2.30; 95% CI: −3.50: −1.09]; however, this finding was based on one RCT. Thus, this finding is inconclusive.

#### 3.4.3. Impact of Surgical Approach

**Figure 4:**
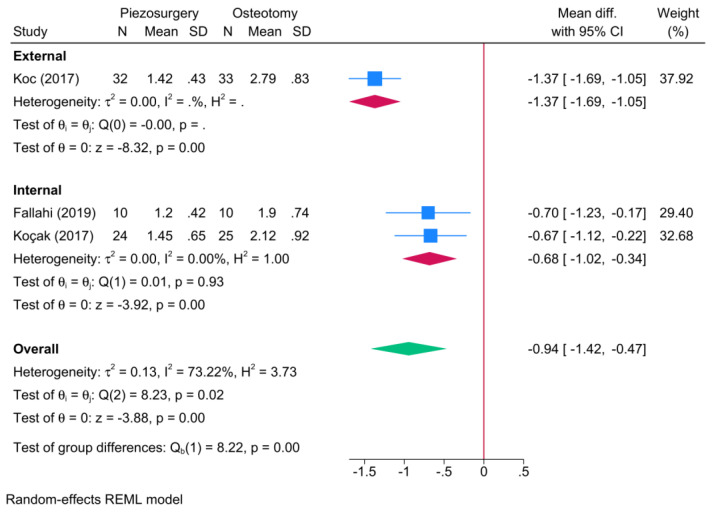
Subgroup analysis of the difference in the degree of postoperative edema between piezosurgery and conventional osteotomy in rhinoplasty patients, stratified by the surgical approach [5,7,8]. Blue squares reflect the effect size of individual studies, while the red rhombi indicate the pooled effect size per each subgroup. The green rhombus highlights the overall pooled effect estimate across all subgroups.

The surgical approach was a significant effect modifier on postoperative edema (*p* = 0.001) (Figure 4). The external approach showed a greater reduction in the score of postoperative edema [one RCT, MD = −1.37; 95% CI: −1.69: −1.05], in favor of piezosurgery, as compared to the internal osteotomy approach [two RCTs, MD = −0.68; 95% CI: −1.02: −0.34]. 

#### 3.4.4. Impact of Edema Grade

The difference in the risk of postoperative edema based on the degree of edema was reported by only two RCTs (Figure S2). Piezosurgery was associated with a greater risk of postoperative edema of Grades 0, 2, and 3. However, it was associated with reduced risk of edema of graded 1 [two RCTs, logOR = −2.28; 95% CI: −3.91: −0.65] and 4. Importantly, all of the other grades were represented by only one RCT. Therefore, no conclusive evidence can be provided in this regard.

### 3.5. Postoperative Ecchymosis

#### 3.5.1. Degree of Ecchymosis

**Figure 5:**
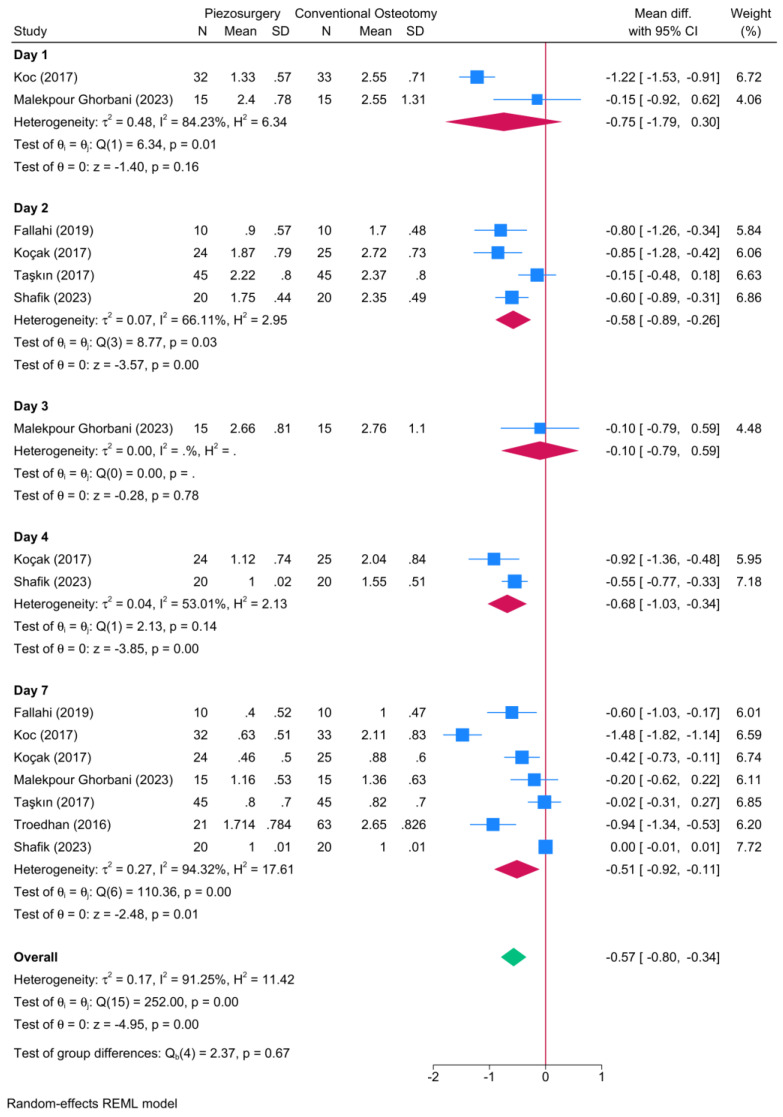
Forest plot showing the difference in the degree of postoperative ecchymosis between piezosurgery and conventional osteotomy in rhinoplasty patients [5,7,8,14,20,21,23]. Blue squares reflect the effect size of individual studies, while the red rhombi indicate the pooled effect size per each subgroup. The green rhombus highlights the overall pooled effect estimate across all subgroups.

Seven RCTs reported data regarding postoperative ecchymosis (Figure 5). A significant reduction in the degree of postoperative ecchymosis was observed in favor of piezosurgery on the second [four RCTs, MD = −0.58; 95% CI: −0.89: −0.26], fourth [two RCTs, MD = −0.68; 95% CI: −1.03: −0.34], and seventh postoperative days [seven RCTs, MD = −0.51; 95% CI: −0.92: −0.11]. No significant different was noted between piezosurgery and conventional osteotomy during the first and third postoperative days. 

#### 3.5.2. Risk of Ecchymosis

The risk of postoperative ecchymosis was reported by four RCTs (Figure S3). The pooled meta-analysis showed a significant reduction in the risk of postoperative ecchymosis in favor of piezosurgery [logOR = −0.83; 95% CI: −1.64: −0.02]. This reduction was only significant at the fourth postoperative day [two RCTs, logOR = −1.07; 95% CI: −1.96: −0.18], with no difference between piezosurgery and conventional osteotomy in the third and fourteenth postoperative days.

#### 3.5.3. Impact of Surgical Approach

**Figure 6:**
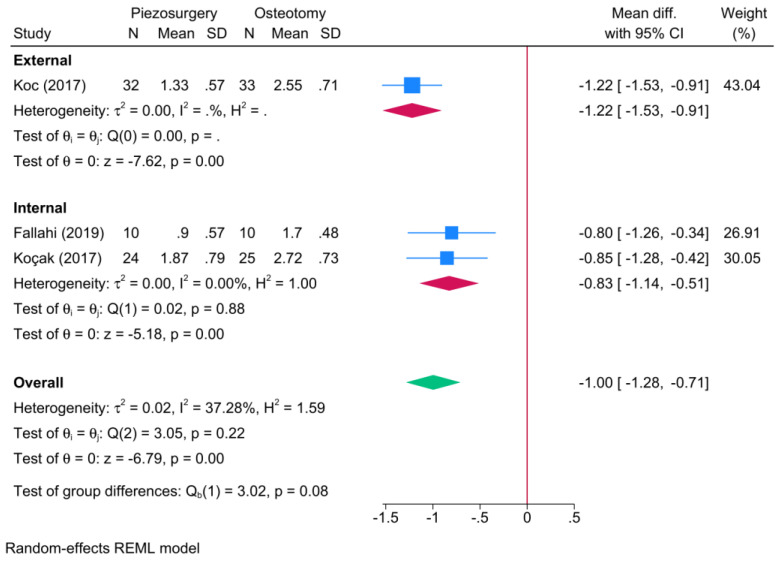
Subgroup analysis of the difference in the degree of postoperative ecchymosis between piezosurgery and conventional osteotomy in rhinoplasty patients, stratified by the surgical approach [5,7,8]. Blue squares reflect the effect size of individual studies, while the red rhombi indicate the pooled effect size per each subgroup. The green rhombus highlights the overall pooled effect estimate across all subgroups.

The surgical approach was not deemed a significant effect modifier on postoperative ecchymosis (*p* = 0.08) (Figure 6), although the external approach showed a greater reduction in the score of postoperative ecchymosis [one RCT, MD = −1.22; 95% CI: −1.53: −0.91], in favor of piezosurgery, compared to the internal osteotomy approach [two RCTs, MD = −0.83; 95% CI: −1.14: −0.51]. 

#### 3.5.4. Impact of Ecchymosis Grade

The difference in the risk of postoperative ecchymosis based on the degree of ecchymosis was reported by two RCTs (Figure S4). No significant difference in ecchymosis was noted between piezosurgery and conventional osteotomy in all ecchymosis grades. Importantly, Grades 0, 1, and 4 were represented by one RCT while Grades 2 and 3 were represented by two RCTs.

### 3.6. Complications

#### 3.6.1. Overall Complications

**Figure 7:**
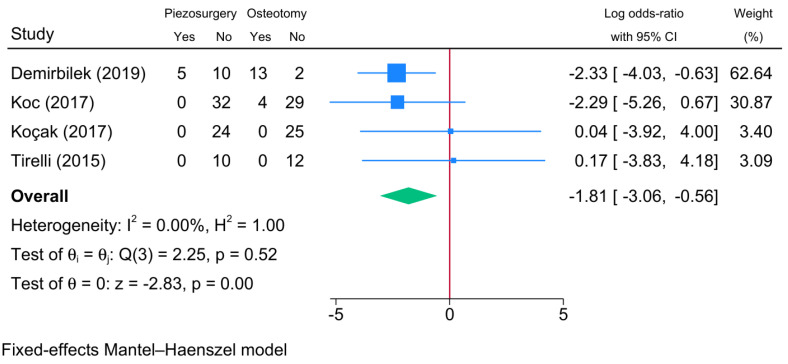
Forest plot showing the difference in the risk of complications between piezosurgery and conventional osteotomy in rhinoplasty patients [5,8,18,22]. Blue squares reflect the effect size of individual studies, and the green rhombus highlights the overall pooled effect estimate across all studies.

Postoperative complications were reported by four RCTs (Figure 7). Overall, piezosurgery was associated with a significant reduction in the risk of complications compared to conventional osteotomy [logOR = −1.81; 95% CI: −3.06: −0.56]. There was no statistical heterogeneity observed [I^2^ = 0%, *p* = 0.52].

#### 3.6.2. Postoperative Hemorrhage

Three RCTs reported the occurrence of postoperative hemorrhage (Figure S5). The pooled meta-analysis showed no significant difference between piezosurgery and conventional osteotomy in the risk of postoperative hemorrhage [logOR = −1.39; 95% CI: −2.95 0.17]. This was maintained across the first, fourth, and seventh postoperative days.

#### 3.6.3. Mucosal Injury

**Figure 8:**
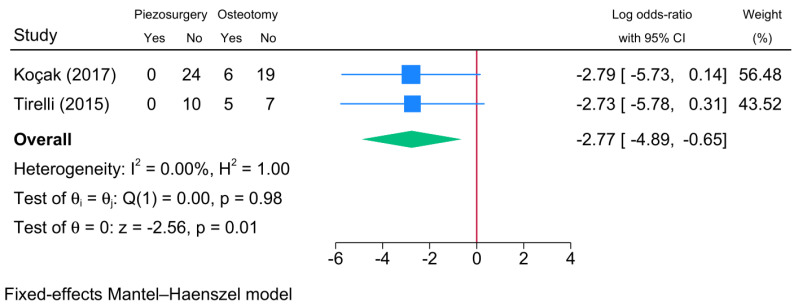
Forest plot showing the difference in the risk of mucosal injury between piezosurgery and conventional osteotomy in rhinoplasty patients [5,22]. Blue squares reflect the effect size of individual studies, and the green rhombus highlights the overall pooled effect estimate across all studies.

The risk of mucosal injury was investigated by two RCTs (Figure 8). The meta-analysis showed a significant reduction in the risk of mucosal injury associated with piezosurgery compared to conventional osteotomy [logOR = −2.77; 95% CI: −4.89: −0.65]. There was no statistical heterogeneity observed [I^2^ = 0%, *p* = 0.98].

### 3.7. Postoperative Pain (VAS Score)

#### 3.7.1. Pain Score

**Figure 9:**
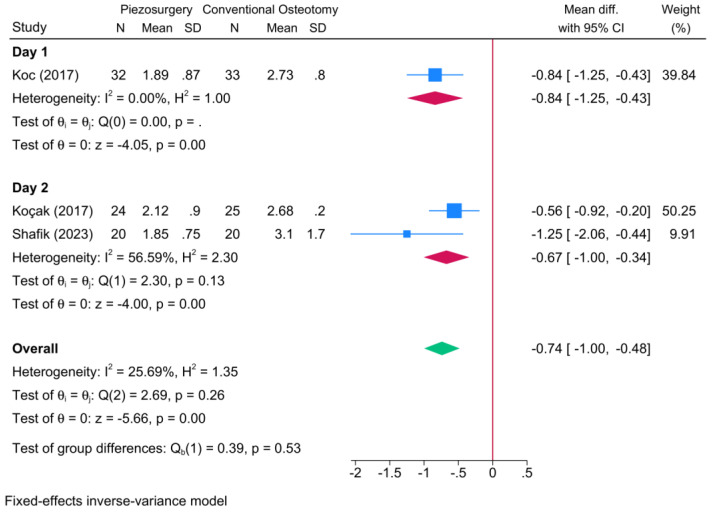
Forest plot showing the difference in pain score between piezosurgery and conventional osteotomy in rhinoplasty patients [5,8,14]. Blue squares reflect the effect size of individual studies, while the red rhombi indicate the pooled effect size per each subgroup. The green rhombus highlights the overall pooled effect estimate across all subgroups.

Two RCTs reported postoperative pain post-rhinoplasty (Figure 9). The meta-analysis revealed that piezosurgery was associated with a significant reduction in the VAS score at the first [one RCT, MD = −0.84; 95% CI: −1.25: −0.43] and second postoperative day [two RCTs, logOR = −0.67; 95% CI: −1.00: −0.34].

#### 3.7.2. Pain Requiring Analgesia (VAS > 3)

**Figure 10:**
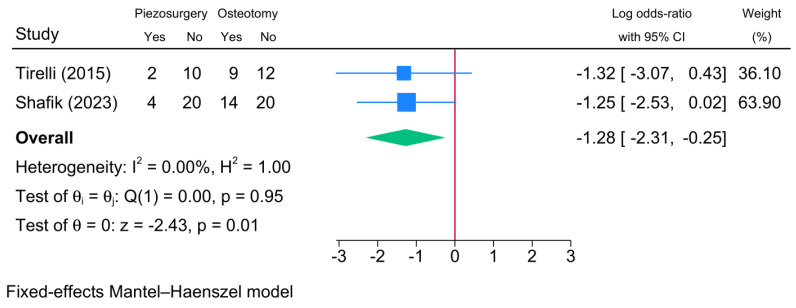
Forest plot showing the difference in pain requiring analgesia between piezosurgery and conventional osteotomy in rhinoplasty patients [14,22]. Blue squares reflect the effect size of individual studies, and the green rhombus highlights the overall pooled effect estimate across all studies.

Two RCTs reported the need for postoperative analgesia (Figure 10). The meta-analysis showed that piezosurgery was associated with a significantly lower risk of postoperative analgesia need compared to conventional osteotomy [logOR = −1.28; 95% CI: −2.31: −0.25]. There was no statistical heterogeneity observed [I^2^ = 0%, *p* = 0.95].

#### 3.7.3. Impact of Surgical Approach

The surgical approach was not an effect modifier on postoperative pain (*p* = 0.70) (Figure S6), although the external approach showed a greater reduction in the VAS score [one RCT, MD = −0.84; 95% CI: −1.24: −0.44], in favor of piezosurgery, compared to the internal osteotomy approach [two RCTs, MD = −0.74; 95% CI: −1.08: −0.39].

### 3.8. Operative Time (Hours)

Four RCTs reported the operative time for both surgeries (Figure S7). The meta-analysis revealed no significant difference between piezosurgery and conventional osteotomy in terms of operative time [MD = 1.50 h, 95% CI: −1.11: 4.10]. However, a substantial degree of heterogeneity was observed [I^2^ = 98.31%, *p* = 0.00]. The leave-one-out sensitivity analysis revealed no significant change in the reported estimate following the exclusion of each study separately (Figure S8).

## 4. Discussion

Despite the increasing adoption of piezosurgery in rhinoplasty, a clear consensus on its comparative effectiveness and safety against conventional osteotomy has remained elusive. This systematic review and meta-analysis fills this critical gap, providing a detailed evaluation of piezosurgery’s role in enhancing postoperative outcomes in rhinoplasty patients.

Our data suggest a significant reduction in the degree of postoperative edema and ecchymosis on specific days (second, fourth, and seventh postoperative days) when using piezosurgery compared to conventional osteotomy. This could be attributed to the precision and less invasive nature of piezosurgery. However, the lack of significant differences on certain postoperative days indicates a complex interplay of factors influencing recovery. The reduction in postoperative edema and ecchymosis can be attributed to the ultrasonic vibrations of the piezotome, which allow for precise cuts, minimizing damage to surrounding tissues and blood vessels. This precision reduces the inflammatory response, which is a primary cause of edema and ecchymosis [24].

Although our findings support the observation of prior meta-analyses [9,10,11] in the sense that piezosurgery provides a better improvement in edema, ecchymosis, and pain postoperatively. However, the discrepancy stemmed from the time in which improvement was observed. For instance, the review by Tsikopoulos et al. [10] reported the improvement in edema/ecchymosis on Postoperative Days 1, 3, and 7. Meanwhile, the other review by Mirza et al. [9] limited this improvement to Days 2, 3, and 7 postoperatively. In this regard, our study provides more reliable findings since our evidence was based on the analysis of 12 RCTs while prior reviews included only 5 RCTs [9,10].

The impact of the surgical approach, in our study, notably the external approach, demonstrated a more substantial reduction in postoperative edema and ecchymosis with piezosurgery. This finding highlights the potential advantage of piezosurgery in specific surgical contexts. The observed advantage in favor of the external approach can be explained by a number of factors: better precision and accessibility, reduced tissue trauma, and enhanced surgical control. For instance, the external approach in rhinoplasty, also known as the “open” approach, involves a small incision on the columella, the tissue between the nostrils. This approach provides surgeons with direct and comprehensive visibility of the nasal structures [25]. In the case of piezosurgery, this enhanced visibility allows for more precise and controlled application of the ultrasonic tool, potentially leading to more accurate bone cuts and less trauma to adjacent tissues [22]. Additionally, piezosurgery’s ultrasonic vibrations are highly effective in cutting bone while sparing soft tissues. When combined with the external approach, the surgeon has better control and can avoid the unnecessary manipulation of soft tissue, leading to reduced trauma. This minimizes the inflammatory response, which is a key factor in postoperative edema and ecchymosis. Furthermore, the external approach provides a broader surgical field and easier access to the nasal framework [26]. This allows for more precise and deliberate movements with the piezotome, reducing the risk of accidental injuries or excessive bone removal. Such control is particularly beneficial in complex or revision cases where precision is paramount.

In our meta-analysis, piezosurgery’s association with a significant reduction in overall complications and specific complications such as mucosal injury is a noteworthy finding. This might be due to the reduced tissue trauma and more controlled cutting technique in piezosurgery [24]. However, the similarity in postoperative hemorrhage risk between the two methods indicates that factors other than the cutting technique may influence this complication. Moreover, the significant reduction in VAS scores and the need for postoperative analgesia with piezosurgery underscore its potential benefit in enhancing patient comfort and recovery. The lack of an effect modifier in the surgical approach suggests a consistently lower pain experience with piezosurgery across different operative techniques.

The absence of a significant difference in operative time between the two techniques, despite the noted heterogeneity, suggests that piezosurgery provides a greater clinical benefit with comparable operative time to conventional osteotomy. The high heterogeneity might be due to variations in surgical procedures, surgeon expertise, and patient characteristics.

These findings suggest that piezosurgery may offer advantages in terms of reduced postoperative edema, ecchymosis, complications, and pain, which can enhance patient outcomes and satisfaction. Surgeons should consider these benefits, especially in contexts where minimizing tissue trauma is critical. On the other hand, the cost of these procedures should be taken into account. For instance, piezoelectric technology is pricier compared to traditional osteotomy methods, with a piezoelectric osteotome unit costing between EUR 5000 and 7000, and replacement tips costing between EUR 150 and 200 each. Although the expense of this technology might be balanced by the noted decrease in postoperative complications, our study cannot make any conclusions about this since none of the included studies provided economic analyses [27].

### Strengths, Limitations, and Future Research Directions

This review’s strength lies in its comprehensive inclusion of RCTs and the methodological rigor in assessing the risk of bias. The global representation of studies also enhances the generalizability of the findings. However, the presence of some concerns in the “selection of reported results” domain, the varying degree of heterogeneity in some outcomes, and the reliance on a limited number of studies for certain comparisons (e.g., postoperative pain) may limit the conclusiveness of these findings. In addition, some outcomes were reported by only one study such as nasal airflow and odor function; no difference was noted between both surgical techniques [16]. Given the fact that studies on efficacy are completely lacking, these outcomes need to be further investigated in future research.

Although our findings confirm the observations made by previous reviews (superiority of piezosurgery in terms of edema and ecchymosis), our findings are more conclusive given the larger sample size. The added value of our study lies in examining other less commonly investigated outcomes, such as postoperative complications, pain and the need for analgesia, and operative time. We also investigated the potential role of the surgical approach on reported outcomes. That being said, the certainty of evidence remains low given the high heterogeneity and the questionable methodological quality of the included studies. Remarkably, main outcomes like edema and ecchymosis were subjectively evaluated among included studies, increasing the likelihood of measurement bias. Factors like patients’ age, gender, baseline nasal anatomy, outcome measures, and comorbid conditions could confound the observed effects, and they were not adequately controlled in the included studies. Unfortunately, we could not perform a meta-regression analysis to determine potential sources of heterogeneity due to the incomplete reporting of patients’ data stratified by the intervention type in the majority of included studies (7 out of 12 studies). That being said, the subgroup analysis revealed that the surgical technique (external or internal) accounted, to some extent, for the observed heterogeneity in outcomes including postoperative edema and ecchymosis as well as operative time.

Our meta-analysis is hypothesis-generating, highlighting current gaps in the available evidence and the directions of future research. Further studies should focus on (1) the long-term comparison between both techniques beyond the one-week frame, (2) a standardized reporting of operative techniques and outcomes, (3) patient-reported outcomes satisfaction levels, and quality of life aspects, (4) the cost-effectiveness of piezosurgery versus conventional osteotomy, and (5) the effect modifying role of the surgical approach on clinical outcomes.

## 5. Conclusions

This systematic review and meta-analysis reveal that piezosurgery, in comparison to conventional osteotomy in rhinoplasty, offers significant benefits in reducing postoperative edema, ecchymosis, complications, and pain, without extending operative time. These findings have important implications for surgical practice in rhinoplasty, advocating for a more nuanced selection of surgical techniques based on specific patient needs and operative goals.

## Figures and Tables

**Table 1:** A summary of the inconsistencies of reporting by available systematic reviews in the literature [9,10,11]. The arrow looking down indicates a significant reduction in the outcome. POD: postoperative day; RCT: randomized controlled trial; ROB: risk of bias; SR/MA: systematic review/meta-analysis; NS: non-significant.

SR/MA	Date of Search	Sample	ROB	Ecchymosis		Edema	Pain	
Tsikopoulos et a ORL	2019	5 RCTs	4 low; 3 unclear	POD 3: POD 7:	↓(4 RCTs) ↓(4 RCTs)	POD 3; POD 7:	↓(4 RCTs) ↓(4 RCTs)	POD 3: ↓(2RCTs)
Mirza et al. The Laryngoscope	2019	6 RCTs	7 high	POD (2/3) POD 7:	↓(5 RCTs) ↓(5 RCTs)	POD 3/4 POD 7:	↓(5 RCTS) ↓(5 RCTS)	POD 2: ↓(2RCTB)
Khajuria et al. PRS Global Open	2022	9 RCTs	4 high; 5 unclear	POD 2: POD 3: POD 4: POD 7:	↓(2 RCTs) ↓(2 RCTs) ↓(1 RCT) ↓(4 RCTS)	POD 2: POD 3: POD 4: POD 7:	↓(2 RCIs) ↓(2 RCTs) Ns(1 RCT) ↓(4 RCTs)	POD 1: ↓(1 RCT) POD 2:↓(2 RCTs) POD 3:↓(1 RCT) POD 7:↓(1 RCT)

**Table 2:** The characteristics of included studies comparing piezosurgery to conventional osteotomy in rhinoplasty.

Author (YOP)	Country	Design	Sample (OS)		Gender (Male)		Age Mean (SD)		Internal Approach		External Approach		Osteotomy Type			Instrument	
			PZ	CO	PZ	CO	PZ	CO	PZ	CO	PZ	CO	PZ	CO	Type	PZ	CO
Aydoğdu (2020) [16]	Turkey	RCT	36	36	Both = 30		Both = 28.1 (6.5)		0	0	36	36	36	36	Lateral	Woodpecker piezosurgery unit SDT-E702	A 2 mm-wide chisel
Demirbilek (2019) [18]	Turkey	RCT	15	15	6	3	28.46 (8.08)	29.86 (8.47)	-	-	-	-	15	15	Lateral and median	Woodpecker piezosurgery unit SDT-E702	4 mm sharp osteotomes
Fallahi (2019) [7]	Iran	RCT	10	10	4	4	24.9 (2.38)	24 (1.63)	10	10	0	0	10	10	Lateral	Principal micro-saw OT7	Double-guarded straight osteotome
Ghavimi (2018) [19]	Iran	RCT	33	33	17	28	-	-	-	0	-	33	33	33	Lateral	Viosurg device from NSK Company with a lateral osteotomy pen	2-mm osteotome
Koc (2017) [8]	Turkey	RCT	32	33	Both = 29		23.6 (5.71)	24.1 (5.72)	-	-	-	33	32	33	Lateral	Piezosurgery Flex medical device	-
Koçak (2017) [5]	Turkey	RCT	24	25	Both = 17		28.5 (3)	25.7 (5.4)	24	25	0	0	13–11	9–16	Transverse with low-to-low lateral—low-to-high lateral	NSK VarioSurg 3 device tips	3 mm guided curved osteotome
Malekpour Ghorbani (2023) [20]	Iran	RCT	15	15	7		Both = 26.6 (5.72)		-	-	-	-	15	15	Lateral	Piezo scalpel OT7S-3	-
Taşkın (2017) [21]	Turkey	RCT	45	45	34		Both = 25.6 (5.6)		-	-	-	-	45	45	Median-oblique and lateral	VarioSurg 3 piezosurgery unit	2 mm guarded, straight osteotome
Tirelli (2015) [22]	Italy	RCT	10	12	10		-	-	0	0	10	12	10	12	Lateral	MT1S-10 tip of the PMD	2-mm wide osteotome
Hashemi (2023) [17]	USA	RCT	31	31	7		Both = 28.43 (7.77)		-	-	-	-	31		Lateral (low-to-low)	The Mectron Piezosurgery	Straight guarded osteotome
Troedhan (2016) [23]	Austria	RCT	21	63	-	-	Both = 44 (11)		-	-	-	-	-	-	-	Piezotome I or II	Chisels and rasps
Shafik (2023) [14]	Egypt	RCT	20	20	6	4	Both = 33.34 (10.23)		-	-	-	-	-	-	Lateral and medial	Piezo medical device with an MT-1 insert tip from	-

YOP: year of publication; RCT: randomized controlled trial; OS: numbers were provided per operated side; SD: standard deviation; PZ: piezosurgery; CO: conventional osteotomy; USA: United States of America.

## Data Availability

The dataset analyzed in this research paper can be made freely available upon a reasonable request to the corresponding author (Alaa Safia).

## Supplementary Materials

The following supporting information can be downloaded at: https://www.mdpi.com/article/10.3390/jcm13133635/s1, Table S1: The criteria employed in searching relevant databases; Figure S1: Forest plot showing the difference in the risk of postoperative edema between piezosurgery and conventional osteotomy in rhinoplasty patients; Figure S2: Subgroup analysis of the difference in the degree of postoperative edema between piezosurgery and conventional osteotomy in rhinoplasty patients, stratified by edema grade; Figure S3: Forest plot showing the difference in the risk of postoperative ecchymosis between piezosurgery and conventional osteotomy in rhinoplasty patients; Figure S4: Subgroup analysis of the difference in the degree of postoperative edema between piezosurgery and conventional osteotomy in rhinoplasty patients, stratified by ecchymosis grade; Figure S5: Forest plot showing the difference in the risk of postoperative hemorrhage between piezosurgery and conventional osteotomy in rhinoplasty patients; Figure S6: Subgroup analysis of the difference in pain score between piezosurgery and conventional osteotomy in rhinoplasty patients, stratified by the surgical approach; Figure S7: Forest plot showing the difference in operative time between piezosurgery and conventional osteotomy in rhinoplasty patients; Figure S8: Leave-one-out sensitivity analysis of operative time between piezosurgery and conventional osteotomy in rhinoplasty patients.
