# Supplementary material for: Is Piezosurgery Associated with Improved Patient Outcomes Compared to Conventional Osteotomy in Rhinoplasty? A Systematic Review and Meta-Analysis of RCTs
Source: J Clin Med. 2024 Jun 21;13(13):3635. doi: 10.3390/jcm13133635 (PMC11242129; doi:10.3390/jcm13133635)
Supplement: Supplementary file 1 [file jcm-13-03635-s001.zip › Table S1.pdf]

Table S1. The criteria employed in searching relevant databases

Database	No	Search Query	Results
**PubMed**			
	#1	Septorhinoplasty[tiab] OR rhinoplast*[tiab] OR "Rhinoplasty"[Mesh] OR septorhinoplasty[tiab]	12819
	#2	Piezosurgery[tiab] OR piezo-surgery[tiab] OR “piezo surgery”[tiab] OR “piezo-electric”[tiab] OR piezoelectric[tiab] OR piezo[tiab] OR "Piezosurgery"[Mesh] OR piezotome[tiab]	16989
	#3	Osteotom*[tiab] OR "Osteotomy"[Mesh]	56145
	#4	random*[tiab] OR "Randomized Controlled Trial" [Publication Type]	1603749
	#5	#1 AND #2 AND #3 AND #4	16
**Scopus**			
	#1	TITLE-ABS-KEY (rhinoplast*) OR TITLE-ABS-KEY (septorhinoplast*)	13842
	#2	TITLE-ABS-KEY (Piezosurgery) OR TITLE-ABS-KEY (piezo-surgery) OR TITLE-ABS-KEY (“piezo surgery”) OR TITLE-ABS-KEY (“piezo-electric”) OR TITLE-ABS-KEY (piezoelectric) OR TITLE-ABS-KEY (piezo) OR TITLE-ABS-KEY (piezotome)	172874
	#3	TITLE-ABS-KEY (Osteotom*)	71228
	#4	TITLE-ABS-KEY (random*)	3211045
	#5	#1 AND #2 AND #3 AND #4	17
**Web of Science**			
	#1	AB=rhinoplast* OR AB=septorhinoplast*	4334
	#2	AB=Piezosurgery OR AB=piezo-surgery OR AB=“piezo surgery” OR AB=“piezo-electric” OR AB=piezoelectric OR AB=piezo OR AB=piezotome	106739
	#3	AB=Osteotom*	28584
	#4	AB=random*	2059300
	#5	#1 AND #2 AND #3 AND #4	10
**CENTRAL**			
	#1	rhinoplast* OR septorhinoplast*	721
	#2	Piezosurgery OR piezo-surgery OR “piezo surgery” OR “piezo-electric” OR piezoelectric OR piezo OR piezotome	754
	#3	Osteotom*	2809
	#4	random*	1369265
	#5	#1 AND #2 AND #3 AND #4	15
**Clinicaltrials.gov**			
	Condition/disease	rhinoplasty	-
	Other terms	osteotomy	-
	Intervention/treatment	piezosurgery	-
	Total	Filter applied: with results	0

**Google Scholar**			
	With all of the words	Rhinoplasty osteotomy random	-
	With the exact phrase	-	-
	With at least one of the words	Piezosurgery pizeo pizeo-surgery piezoelectric piezo-electric piezotome	-
	Total	-	200

CENTRAL: Cochrane Central Register of Controlled Trials
