# Supplementary figures and images for: Is Piezosurgery Associated with Improved Patient Outcomes Compared to Conventional Osteotomy in Rhinoplasty? A Systematic Review and Meta-Analysis of RCTs
Source: J Clin Med. 2024 Jun 21;13(13):3635. doi: 10.3390/jcm13133635 (PMC11242129; doi:10.3390/jcm13133635)

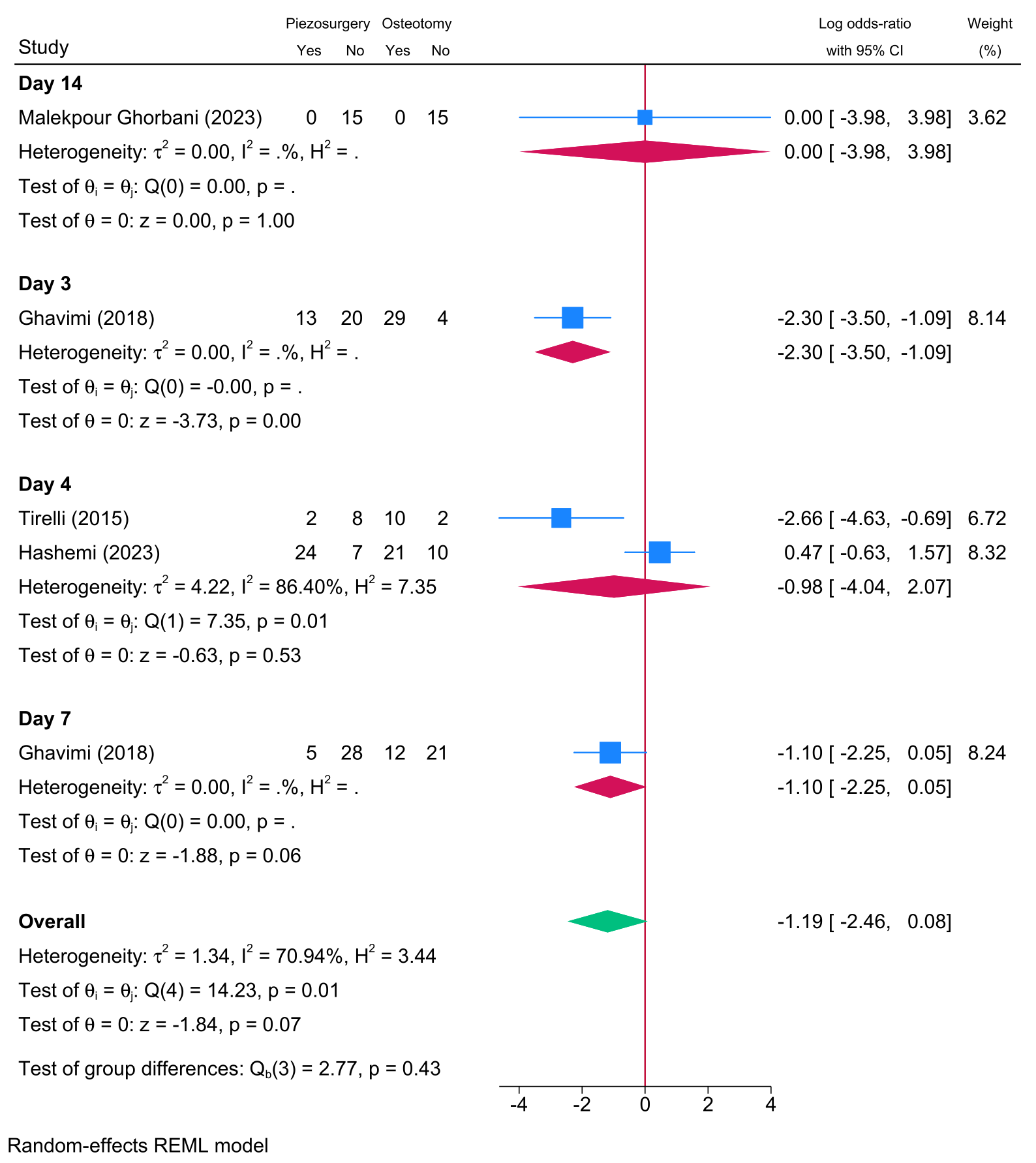

Supplement: Supplementary file 1 [file jcm-13-03635-s001.zip › Figure S1.tiff]

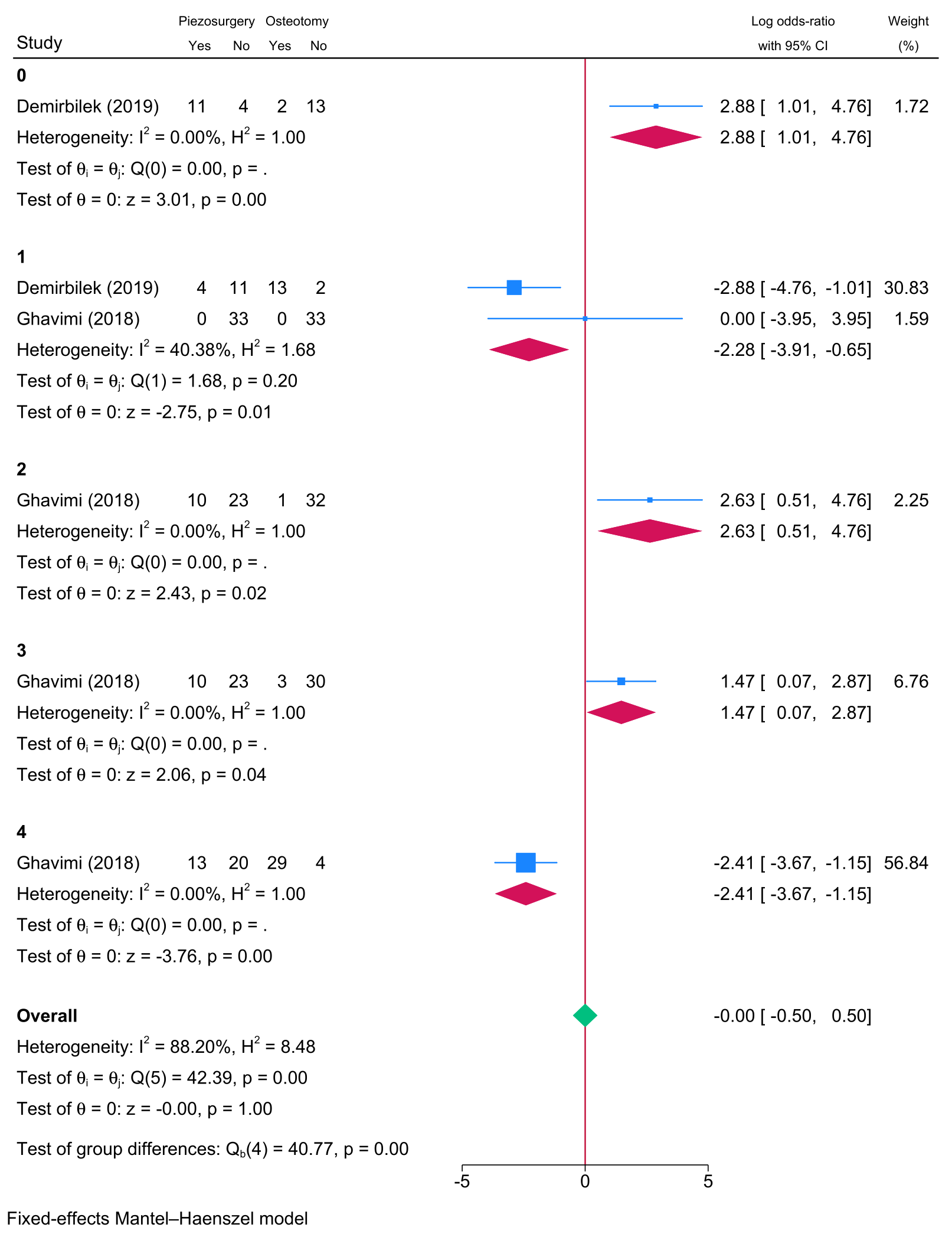

Supplement: Supplementary file 1 [file jcm-13-03635-s001.zip › Figure S2.tiff]

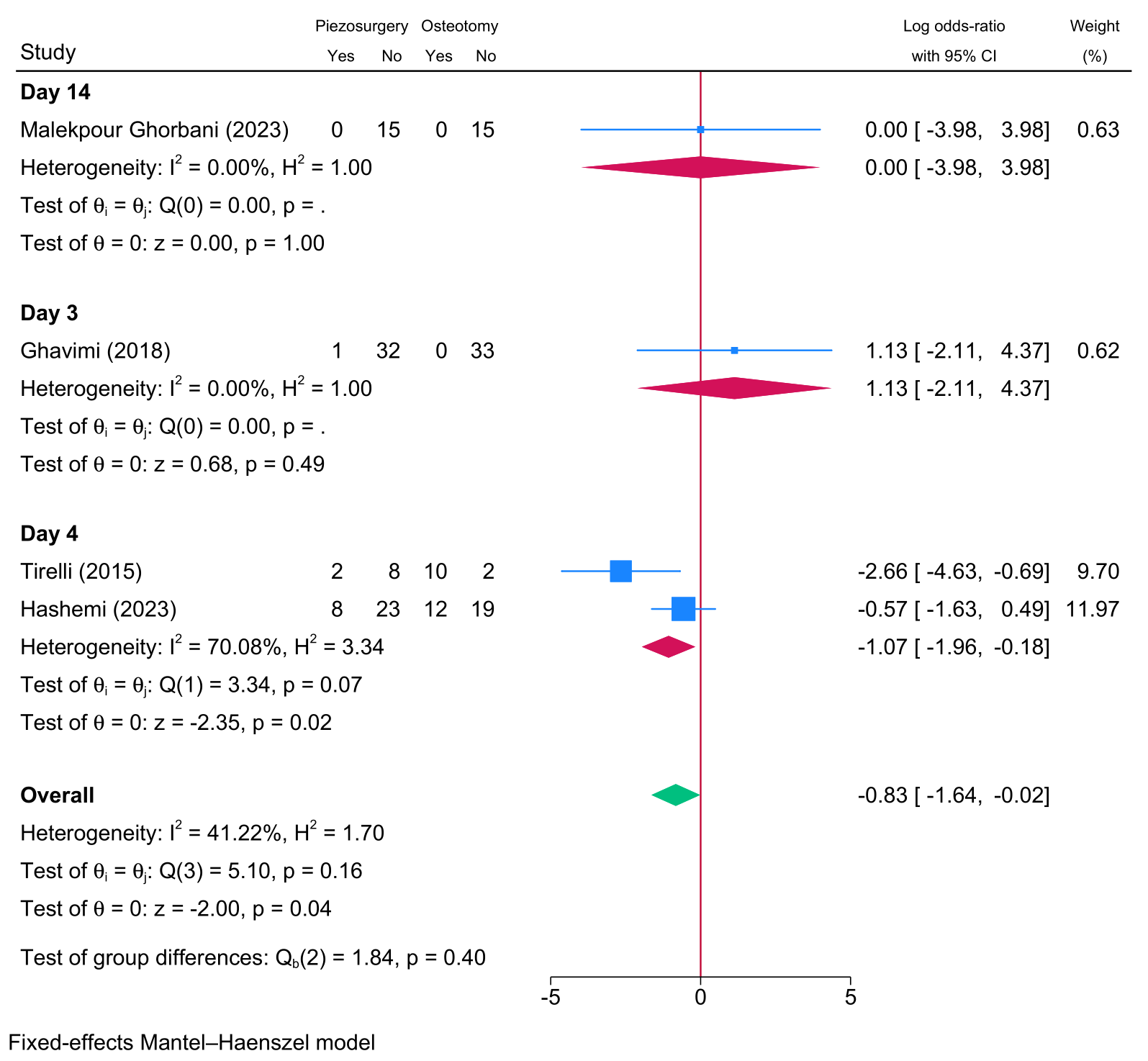

Supplement: Supplementary file 1 [file jcm-13-03635-s001.zip › Figure S3.tiff]

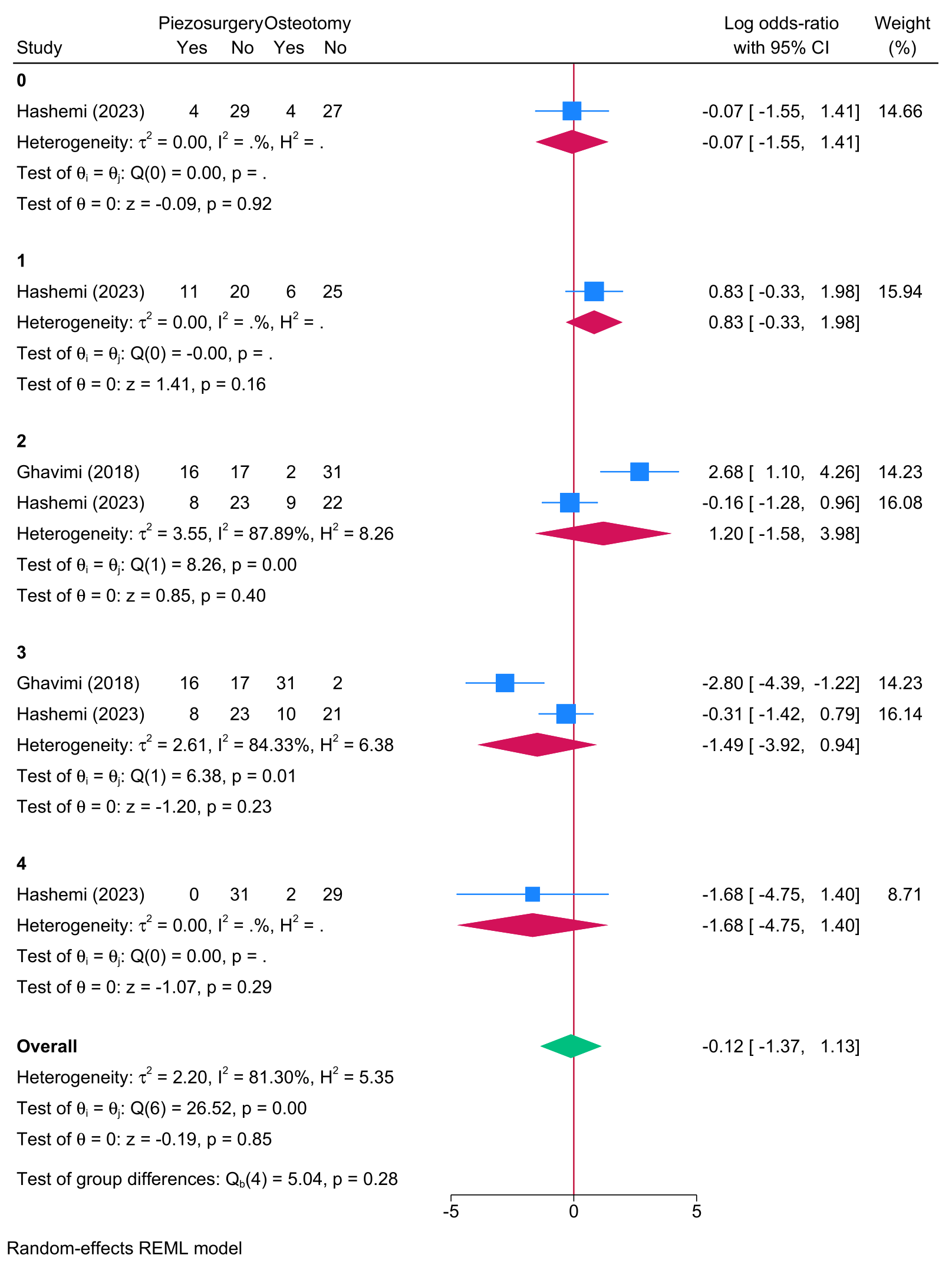

Supplement: Supplementary file 1 [file jcm-13-03635-s001.zip › Figure S4.tiff]

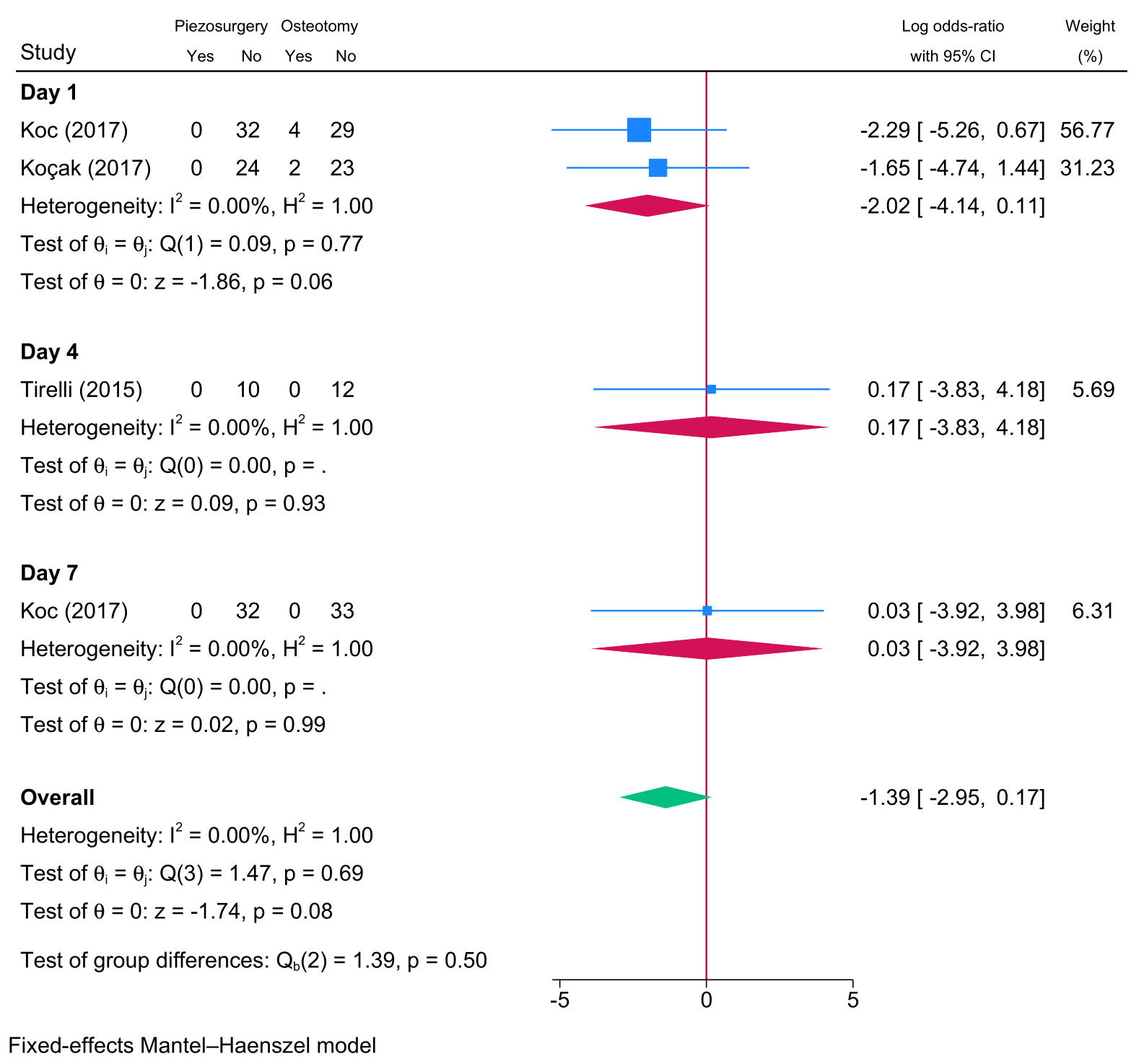

Supplement: Supplementary file 1 [file jcm-13-03635-s001.zip › Figure S5.tiff]

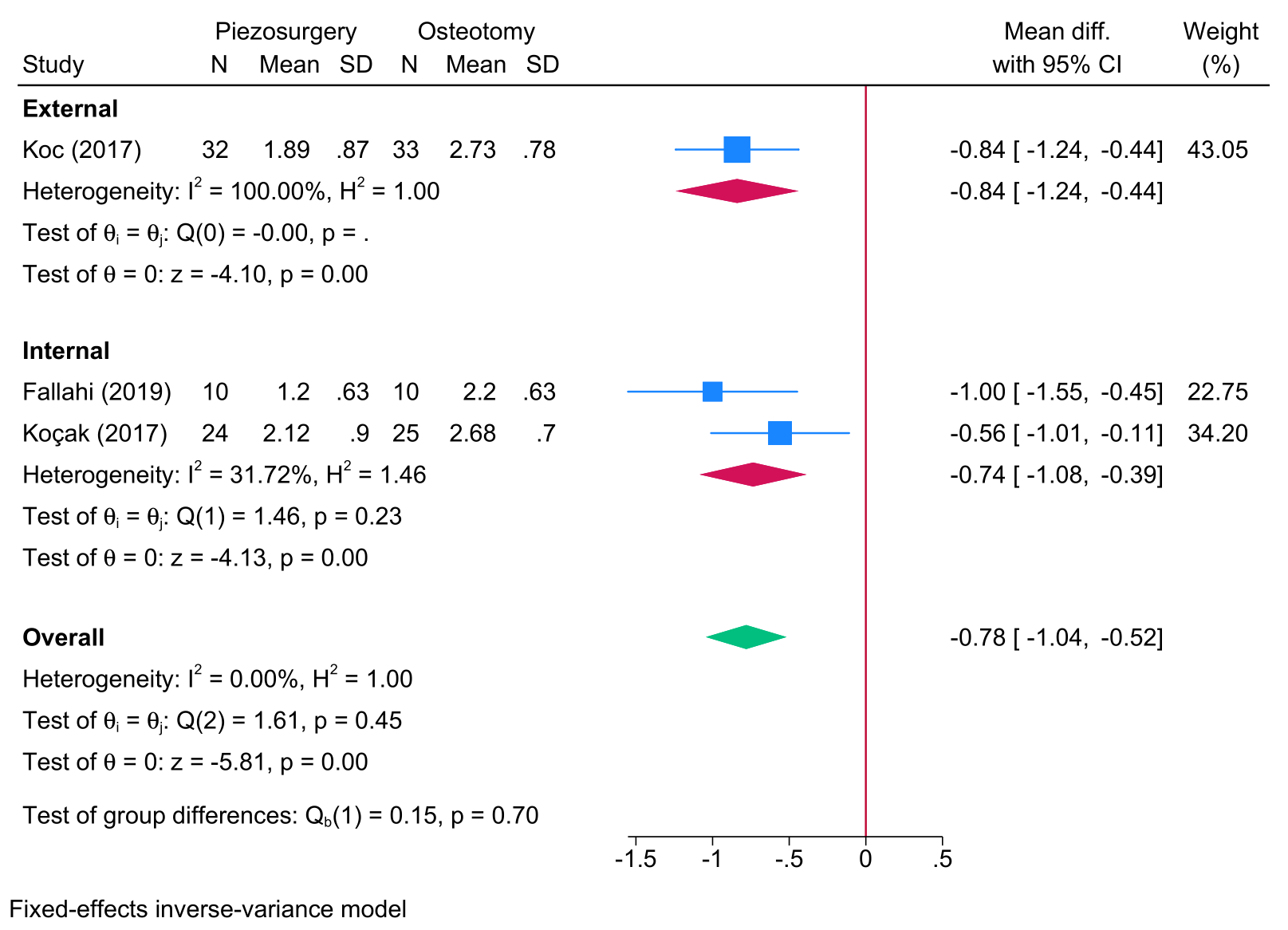

Supplement: Supplementary file 1 [file jcm-13-03635-s001.zip › Figure S6.tiff]

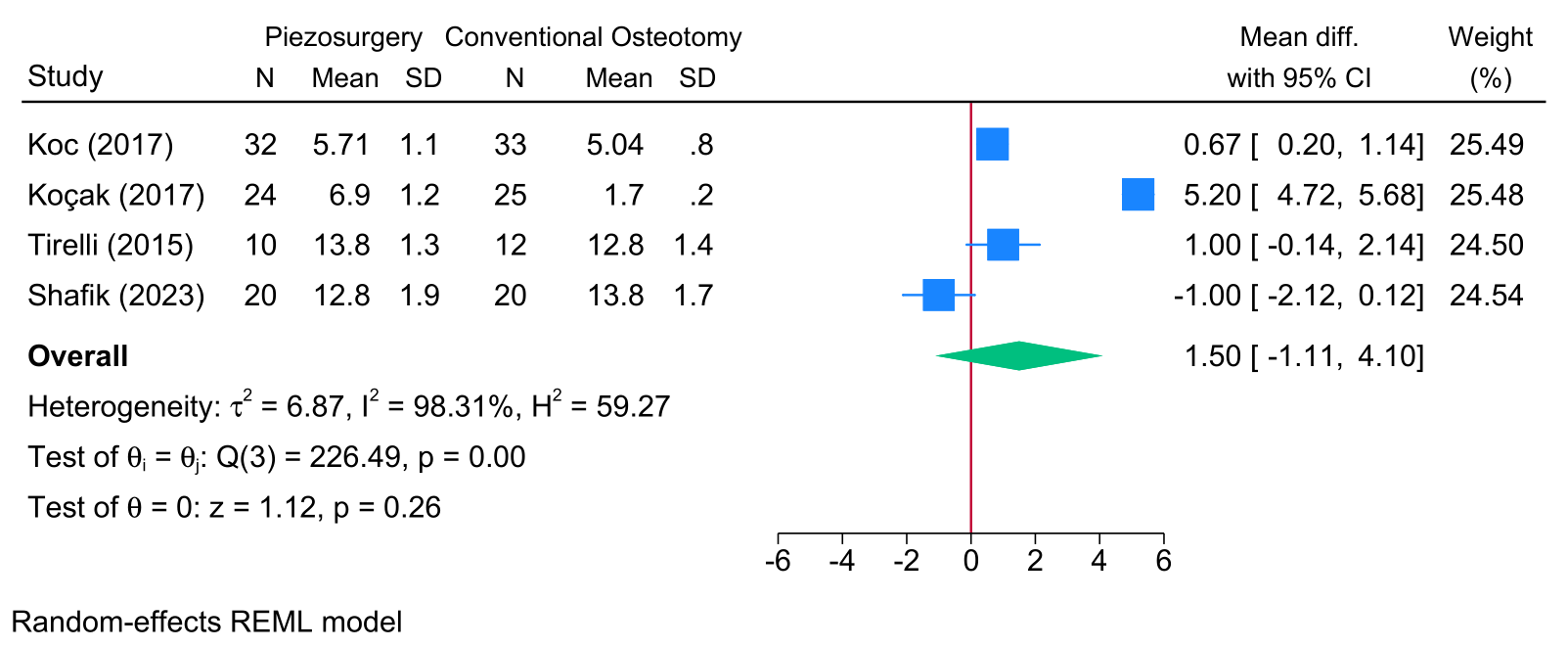

Supplement: Supplementary file 1 [file jcm-13-03635-s001.zip › Figure S7.tiff]

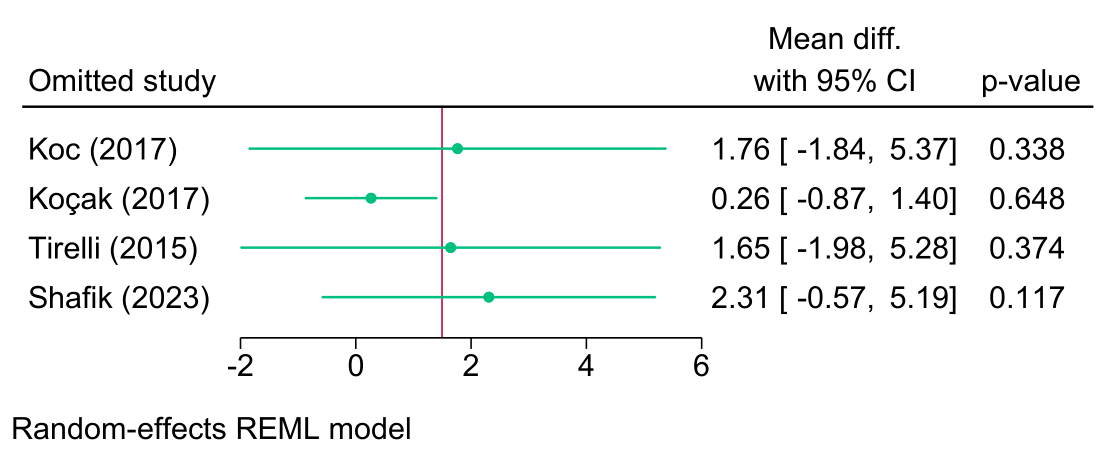

Supplement: Supplementary file 1 [file jcm-13-03635-s001.zip › Figure S8.tiff]
